# To Discover the Efficient and Novel Drug Targets in Human Cancers Using CRISPR/Cas Screening and Databases

**DOI:** 10.3390/ijms222212322

**Published:** 2021-11-15

**Authors:** Iichiroh Onishi, Kouhei Yamamoto, Yuko Kinowaki, Masanobu Kitagawa, Morito Kurata

**Affiliations:** Department of Comprehensive Pathology, Graduate School of Medicine, Tokyo Medical and Dental University, Tokyo 113-8510, Japan; kypth2@tmd.ac.jp (K.Y.); endpth2@tmd.ac.jp (Y.K.); masa.pth2@tmd.ac.jp (M.K.)

**Keywords:** CRISPR screening, synthetic lethality, database

## Abstract

CRISPR/Cas has emerged as an excelle nt gene-editing technology and is used worldwide for research. The CRISPR library is an ideal tool for identifying essential genes and synthetic lethality targeted for cancer therapies in human cancers. Synthetic lethality is defined as multiple genetic abnormalities that, when present individually, do not affect function or survival, but when present together, are lethal. Recently, many CRISPR libraries are available, and the latest libraries are more accurate and can be applied to few cells. However, it is easier to efficiently search for cancer targets with their own screenings by effectively using databases of CRISPR screenings, such as Depmap portal, PICKLES (Pooled In-Vitro CRISPR Knockout Library Essentiality Screens), iCSDB, Project Score database, and CRISP-view. This review will suggest recent optimal CRISPR libraries and effective databases for Novel Approaches in the Discovery and Design of Targeted Therapies.

## 1. The Evolution and Usefulness of Random Mutagenesis in Cancers

Cancer biology has been well developed mainly by deciphering the sequence of genes, such as TCGA (the Cancer Genome Atlas Program: https://www.cancer.gov/tcga (accessed on 24 October 2021)), and analyzing the functions of the proteins encoded by the genes [1,2]. Artificial mutations are introduced into the targeted gene to analyze the function of a gene; its function is lost or gained, and the targeted gene function is identified or verified by observing the biological phenotypes. In addition, the randomly mutated cultured cells can identify the potential factors contributing to proliferation or cell death. Specifically, random mutagenesis has been induced using carcinogens by retroviruses, transposons, and short hairpin RNA (shRNA) libraries [3,4]. The shRNA library is a suitable system for knockdown (KD) of specific genes and for screening elements responsible for specific phenotypes. Screening using the shRNA library has produced fruitful results in finding effective targets of cell proliferation in cancers and has greatly contributed to scientific developments [4,5,6]. However, the shRNA library contains many candidates, including many nonfunctional candidates, and can hardly detect essential genes, as they are missed along with cell death compared to new CRISPR screenings [7]. The shRNA library is difficult to use for screening phenotype differences by upregulation of potentially responsible elements, which may be caused by retroviruses and transposons.

Developing a gene-editing method using the bacterial clustered, regularly interspaced short palindrome repeats (CRISPR)/Cas9 system is a huge breakthrough in biological fields [8]. The protospacer adjacent motif sequence (PAM) is a sequence conserved in almost all genes, and the design of a guide RNA (gRNA) directly under the PAM sequence induces double- or single-strand breaks in DNA at the gRNA target site. Subsequent non-homologous recombination or homologous recombination-type repair causes specific mutations at the same site. Specific mutations and frameshifts can cause a loss of function of the protein. The CRISPR/Cas9 system allows us to easily introduce mutations into targeted molecules with high efficiency. In summary, the CRISPR/Cas9 system can (1) design any target site adjacent to the PAM region; (2) modify genes at the DNA level; (3) make mutations occur in the nucleus; and thus (4) introduce permanent modification at any DNA sites. Two groups can be used to apply the CRISPR/Cas9 system to human genes [9,10]. By introducing only bacterial Cas9 and gRNA, which are designed by preference, to human cell lines, human genes can be knocked down. Subsequently, with the development of nuclease inactive Cas9 (dCas9), which knocks out target genes and improves the regulation of transcriptional activities for target genes with transcriptional regulators such as VP64 and KRAB [11,12], the CRISPR system has been used to enhance gene expression in a gRNA binding-sequence-specific and transcription-start-site-specific manner. The CRISPR-dCas9-VP64 (CRISPR activation: CRISPRa) system is used to increase gene expression [11,13,14,15], while CRISPR-dCas9-KRAB (CRSIPR inhibition: CRISPRi) is used to decrease gene expression [11].

CRISPR libraries for random mutagenesis were published simultaneously in *Science* by two groups: Shalem et al. and Wang et al. in 2013 [16,17]. Shalem et al. designed 64,800 gRNAs and 73,000 gRNAs were designed by Wang et al. for 18,080 genes. Random mutagenesis using conventional methods, such as retroviruses and transposons, has been used to identify target molecules and drug discovery in cancer and other diseases. Adding the CRISPR library enables us to specifically analyze the human genome-wide loss and gain of functions. Furthermore, it is difficult to screen the function of long non-coding RNAs (lnc RNAs) that do not encode proteins by CRISPR knock-out library or CRISPR knock-out, but it is possible to analyze them by CRISPRa and CRISPRi libraries [18]. Various CRISPR-pooled libraries, such as Addgene, are published and commercially available from repository institutions. In addition, third-generation CRISPR libraries with higher specificity are now available due to emerging gRNA design algorithms. Table 1 and Table 2 show the libraries using lentivirus vectors for human subjects. Various libraries are available for knock-out, activation, and inhibition, and different organisms, such as mice and humans. The CRISPR library is a useful method for identifying cell survival (essential) genes, proliferation factors, and drug resistance under anticancer drug administration. Guide RNAs retained in the cells after the screening conditions are analyzed to identify genetic mutations (upregulation of oncogenes, downregulation of tumor suppressor genes, etc.). Negative selection is suitable for identifying the gRNAs, which disappear with dead cells, and are analyzed by subtracting the gRNAs of surviving cells from the original library. Thus, identifying gRNAs is equal to essential genetic mutations that are detrimental to survival [19].

## 2. Application of CRISPR Libraries, Optimization of gRNA, and Efficient Next-Generation Libraries

Before applying gRNA to human cells, the Cas9 nuclease must be introduced to mammalian cell lines. *Streptococcus pyogenes* Cas9 nuclease is usually used. A lentiviral vector backbone is usually used because of its high transduction rate, easy manipulation, and permanent expression of gRNA after built-in cell line DNA. One plasmid system includes a gRNA library and Cas9 plasmids in one same plasmid and is easy to be handled. However, in the multiple vector system, the vector size is generally small and has a relatively high efficiency of the transduction ratio. For library screening, transduction efficiency is important, and multiplicity of infection (MOI) is an important parameter. MOI can be defined as the ratio of the number of virus particles to the number of target cells. For CRISPR screening, the MOI usually ranges in 0.4–1.0. To maintain the diversity of gRNAs in CRISPR libraries in the screening, scientists should use numerous cells. For example, one hundred times coverage is expected for 64,800 gRNAs, and 6.48 × 10^6^ cells are needed. Indeed, transduction efficiency is not 100% and needs more cells. Therefore, reducing the number of gRNAs has a benefit for precious cells, such as primary culture cells.

The GeCKO library v2, published in 2014, has been the KO library [22]. Initially, the number of gRNAs were set at approximately six per target gene, which was difficult to ensure sufficient coverage regarding primary cultured cells or cell lines with low transduction efficiency. Recently, Doench et al. designed optimized sgRNAs for *S. pyogenes* Cas9 using a gRNA design algorithm method and developed the Brunello library with three to four sgRNAs per target gene but improved on-target and off-target effects [23]. The Brunello library has improved on-target efficiency and off-target effect despite having only four sgRNAs per target gene, and is particularly useful when numerous cells cannot be prepared, such as in primary cultures. Toronto KnockOut Version 3 (TKOv3) has the same improvements in gRNAs as above, with four gRNAs per gene, improving the on-target and off-target effects compared to Toronto KnockOut Version 1 (TKOx1) [29].

In the KO library, essential genes may be missed, along with cell death, because they play key roles in cell survival. In addition, genes with high copy numbers may mimic essential genes because of the potential for accumulation of dsDNA cleavage, which may cause cell death. In such cases, CRISPRi is useful for loss of function analysis. However, the reliable loss of function was inferior to the KO library. Doench et al. used the FANTOM database to design sgRNAs with an optimal window for the TSS and designed the library [33]. In the Dolcetto library, A: the top three ranked sgRNAs and B: the next three ranked sgRNAs are designed. The Dolcetto library can sort essential and non-essential genes with the same accuracy as the Brunello KO library and outperforms the conventional CRISPRi library with 3 sgRNAs compared to the conventional 10 sgRNAs per gene [23]. In the future, using this library with a CRISPR KO library and different modalities will be useful for narrowing down candidate genes in exon portions. It will also increase the accuracy of the analysis of non-coding lesions, which may induce new discoveries.

CRISPRa is suitable for identifying genetic mutations that favor cell survival and proliferation (upregulation of oncogenes, etc.) and drug resistance factors under anticancer drug administration (synthetic rescue) by analyzing the gRNAs of cells that remain or proliferate under screening conditions. Using CRISPRa (and also CRISPRi), which targets transcription factors, has enabled a comprehensive analysis of the lnc RNA region, which has been difficult to analyze in the past. Bester et al. searched for factors involved in Ara-C resistance, a therapeutic drug for acute myeloid leukemia, by combining a comprehensive cell line database and CRISPRa for non-coding regions (14,701 regions) (positive selection) [23,34]. Furthermore, as a new finding, they identified that the GAS6-AS2 lncRNA contributes to Ara-C resistance by activating the GAS6/TAM pathway.

The conventional and widely used CRISPRa library—a Synergistic Activation Mediator (SAM)—has a potential problem in the vector of MS2-p65-HSF1, a large vector size, which results in low efficiency of infection and hence low efficiency of activation. To improve the efficiency, two MS2 and two PP7 stem loops were introduced into the Calabrese library on tracrRNAs to facilitate the recruitment of transcription factors by attaching PCP, p65, and HSF complexes [23]. However, in Vemurafenib resistant genes using A375 melanoma cell lines, Calabrese screens revealed substantially more hits, and then the SAM library showed better concordance of sgRNAs targeting the same gene. Additionally, in the secondary screening, the Calabrese library showed a low false discovery rate (FDR), and in the 47 genes with an FDR of <5%; 37 genes were newly found in Calabrese library. In addition, because the CRSIPRa component can be used to design sgRNAs 75–150 bp upstream of TSS to optimize sgRNA design, using Calabrese and the SAM library would search for efficient candidate genes with low FDR [23]. Recently, algorithms for designing gRNAs with higher on-target efficiency and lower off-target effects have been developed, and libraries that allow efficient screening with fewer cells by reducing the number of gRNAs per target gene have been developed.

## 3. CRISPR Screening Focusing on Synthetic Lethality

**Figure 1:**
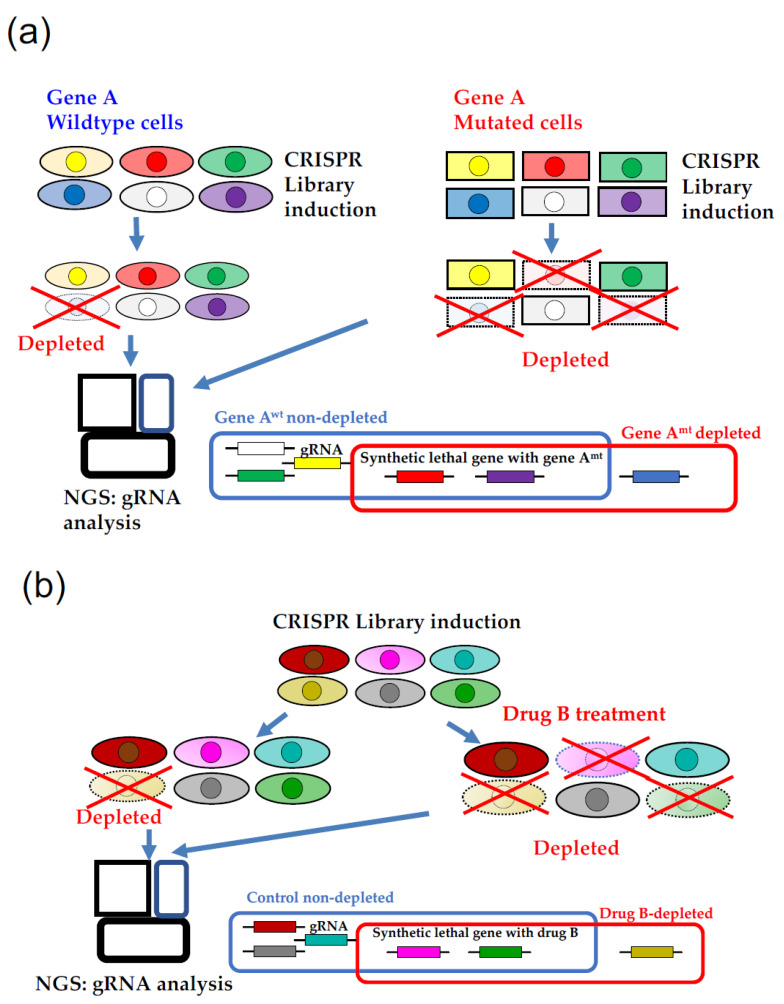
Synthetic lethal research using a CRISPR KO library. (**a**) Synthetic lethal gene detection with gene A using CRISPR (KO) library. Gene A mutated cells (square cells) containing red and purple gRNA induce cell death alongside the culture. Red and purple gRNA are synthetic lethal genes with a gene A mutation. While blue gRNA, which induced cell death in gene A wild type and gene A-mutated cells, is an essential gene for both cells and not a synthetic lethal gene. (**b**) Synthetic lethal gene detection with drug B using CRISPR KO library. Cells containing pink and green gRNA induce cell death under drug B treatment. Pink and green gRNA are synthetic lethal genes with drug B treatment, while ocher gRNA, which induced cell death alongside the culture, is an essential gene for cells and not a synthetic lethal gene.

Recently, the concept of synthetic lethality has gained ground in cancer treatment, and the development of anticancer drugs regarding synthetic lethality is expected [35]. Synthetic lethality refers to the concept that multiple genetic abnormalities or specific gene-targeting agents, when present individually, do not affect function or survival, but when present together, are lethal (Figure 1a,b). For example, in cancer cells with mutations in *BRCA1* or *BRCA2,* which are involved in DNA double-strand breaks, the mutations by themselves are not detrimental to the survival of the cancer cells (rather, the accumulation of genetic mutations is likely to cause clonal evolution), but the administration of a DNA single-strand break repair enzyme inhibitor, PARP inhibitor, results in death. By administering PARP inhibitors, the BRCA-mutated cancer cells are prevented from repairing their DNA, and are made to die. Thus, a good synthetic lethal drug lacks lethal effects on normal tissues and cells, but is lethal to cancer cells with certain mutations.

Synthetic lethality research requiring complete knockdown of gene expression and CRISPR screening, which introduces genome-wide mutations, is well suited for screening for synthetic lethality and novel cancer therapies. Many synthetic lethality CRISPR screenings have been performed (Table 3). CRISPR screening for detecting the candidate genes of synthetic lethal [36,37] and pancreatic cancer cells [38] has recently been reviewed.

In CRISPR KO and CRISPRi, loss of function (LOF) analysis was performed by subtracting the gRNAs of surviving cells from the original library of dead cells (negative selection) (Figure 1a). Thus, in a broad sense, synthetic lethality can be defined as lethality for specific drugs (Figure 1b). This method is suitable for identifying genetic mutations that are detrimental to survival, but the analysis is rather complicated.

We introduce important reports about synthetic lethality using CRISPR screening. *KRAS* mutation, such as KRAS (G13D), is the most frequent cancer driver mutation and an intractable anticancer drug. Yau et al. used the GeCKO v2 library (KO library) for *KRAS* mutant HCT116 colorectal cancer cell lines and transplanted to mice (in vivo xenograft transplantation model) [39]. They found that metabolic pathway components, SUCL2A, NADK, and KHK, were associated with the MAPK signaling. Moreover, focusing on secondary validation sgRNA screen by deep sequence revealed that INO80C was a synthetic lethal partner for *KRAS* mutation. Like the SWI/SNF complex, INO80 is a large multi-subunit complex maintaining genome stability through nucleosome editing, and INO80C is the homolog of the INO80. Thus, INO80C may be a novel target for KRAS mutant tumors.

Steinhart et al. introduced the TKO gRNA library to HPAF-2 cells (*RNF43*-mutant) and identified the Wnt pathway, WLS, CTNNB1, TCF7L2, and LRP5, and Fizzled (FZD) receptor genes, FZD5, WNT7B, and WNT10A, as essential factors for HPAF-2 cells [40]. They showed that proliferation and survival of HPAF-2 cells are selectively dependent on the Wnt pathway.

Abraham et al. employed genome-wide CRISPR screening to characterize the mechanism of action of the transcriptional repressorΔNp63α in SCC [41]. They constructed doxycycline-inducible ΔNp63α depletion in the lung squamous cell line, H226 cells, and applied GeCKOv2 library. CRISPR screening identified small GTPase RHOA as a mediator of proliferation arrest upon ΔNp63α depletion. Moreover, ΔNp63α transcriptionally suppresses TGFB2 expression, and TGFB2 activates RHOA. However, ΔNp63α depletion and neutralization of TGFB2 restore SCC cell proliferation during DNp63a depletion. In short, theΔNp63α-TGFB2-RHOA axis may be a target of lung SCC.

Ferroptosis is a way of cell death, and glutathione peroxidase 4 (GPX4) plays a pivotal regulator role in ferroptosis. GPX4 is upregulated in various tumors, like malignant lymphoma [59], and ferroptosis is a good target for synthetic lethality. Bersuker et al. performed a synthetic lethal CRISPR-Cas9 screen in ferroptosis-resistant human U-2 OS osteosarcoma cells [50]. With the GPX4 inhibitor, RSL3, they treated U-2 OS cells, used a sub-library of single-guide RNAs (sgRNAs) targeting genes related to apoptosis, and revealed that sgRNAs targeting ferroptosis suppressor protein 1 (FSP1) were greatly reduced in cells. Synergizing *FSP1* gene deletion and RSL3 treatment resulted in synthetic lethality. Furthermore, for the H460 cell line (derived from human large cell lung cancer) xenograft mouse models, both *GPX4* and *FSP1* knock-out cells showed rapid death—synthetic lethality.

Recently, Olivieri et al. focused on the DNA damage response induced by anticancer drugs and used the CRISPR library to identify novel genes that are candidates for synthetic lethality with anticancer drugs [37]. RPE1-hTERT p53(-/-) Flag-Cas9 cells (based on primary human retinal pigment epithelial cells [RPE1] cells, in which the hTERT gene was introduced and p53 was knocked out) with the TKO library (TKO v2, TKO v3) were treated with 27 different DNA-damaging agents to identify 890 genes that confer sensitivity or resistance to DNA-damaging agents. They identified chemogenomic networks of DNA damage response: (1) as novel DNA repair genes, they identified ERCC6L2, ELOF1, STK19, and the mechanism by which the guanine quadruplex (G4) ligand; (2) cytotoxicity of the G-quadruplex ligand pyridostatin involves TOP2 trapping; and (3) the mechanism of drug metabolism by TXNDC17 [37]. These findings will reinforce the conventional anticancer drug response.

To find the effective combination of molecular targets and synthetic lethality in a broad sense for cancer treatments, the combination of dual CRISPR screenings were also invented. Indeed, Han et al. invented a CRISPR-based double knock-out (CDKO) system that can provide the efficiency of combinatorial genetic screening with a robust statistical scoring method for calculating genetic interactions. They used 700 × 700 = 490,000 double-sgRNAs directed against 21,321 pairs of drug targets and found that the combination against both BCL2L1 and MCL1 inhibitors was effective for imatinib-resistant leukemic cells [44]. In addition, Gier et al. reported that optimized Cas12a (Cpf1) enables multigene editing from a single RNA transcript and is potentially suited to multiplex editing for combinatorial genetic screening. A dual-crRNA library with 8281 pairwise targeting of 21 epigenetic regulatory domains revealed three synthetic sick interaction pairs; *Brd9&Jmjd6*, *Kat6a&Jmjd6*, and *Brpf1&Jmjd6* [49]. Combining CRISPR screenings is promising, however, maintaining the diversity of numerous gRNAs is challenging. New strategies for dealing with a large number of gRNAs or reducible numbers of gRNAs to cover targeted genes are expected.

## 4. Efficient CRISPR Screening Using the Database to Avoid Pitfalls

In CRISPR screening, many candidate gRNAs are identified, regardless of whether they are functional or not. Many of them may be off-target or deviate from the target phenotype due to unknown incidents during screening. To accurately identify target molecules, it is important to set the MOI below 1.0 and avoid duplication of gRNAs in a single cell. Although, in theory, it is easy to manipulate the MOI, the actual transduction efficiency may change depending on the cell conditions. Therefore, checking the copy numbers of viral integration would be helpful. Moreover, to narrow down the candidate genes, it is imperative to use available databases to facilitate the research. Since the use of the CRISPR library is widespread worldwide, considerable knowledge has been accumulated (Table 4). Using this database can significantly reduce the effort to find real targets.

The DepMap portal (https://depmap.org/portal/ (accessed on 24 Octorber 2021)) integrates the CRISPR knockout screening dataset (Achilles), cell line database (CCLE), and drug susceptibility databases (Profiling Relative Inhibition Simultaneously in Mixtures (PRISM). The Cancer Therapeutics Response Portal (CTRP) provides a comprehensive database of cancer cell vulnerability [60]. Dependent cell lines were identified using the CRISPR library (DepMap 21Q2 Public + Score, CERES) and the RNAi library (Achilles + DRIVE + Marcotte, DEMETER2). For example, by entering a gene name (e.g., KRAS), you can see Dependent Cell Lines (selective or not), Mutations, Enriched Lineages, Expression and Copy Numbers in the CRISPR and RNAi libraries; TopCo-dependencies, Target Tractability (Bioactive Compounds, presence of Druggable Structure, etc.), Description, and links to PubMed and GTEx are available in the Overview. In addition, there are tabs for Perturbation effects, Characterization, and Dataset selection. The database also includes CCLE, PRISM, and CTRP.

Furthermore, the Wellcome Sanger Institute in the U.K. is also developing Project Score as part of its Cancer Dependency Map, intending to use the research results of the CRISPR library to help prioritize new target gene candidates for cancer therapy [61]. The most significant feature of this project is that it targets 18,009 genes in 204 cancer cell lines across 12 different tissues, including lung and colon, and introduces fitness genes (FGs), which are required for cancer cell fitness (growth or survival) by CRISPR-Cas9 screening. Hence, this is where we introduce the FG by separating FGs that are common to all cancer cell lines (pan-cancer core fitness (CF) genes) from FGs that are specific to a particular cancer cell line (cancer-type CF genes). The researchers believe that cancer-type CF genes can be targeted for therapy. The results of this analysis were then correlated with biomarkers (SNVs, CNVs, or microsatellite instability) and the frequency of somatic mutations in the patient’s tumor to define a priority score (0–100). The 497 genes with scores exceeding the threshold were identified as priority target genes, and the genes were further classified into three classes (A, B, and C) based on the evaluation of factors that affected the score, the availability of inhibitors, and clinical trial data. For example, PIK3CA is classified as class A in breast, lung, colorectal, and ovarian cancers, and PIK3CA inhibitors are used in clinical trials for the treatment of PIK3CA-mutated cancers. The BROAD Institute and the Sanger Institute are integrating their CRISPR screening data to create a comprehensive Cancer Dependency Map [65].

The Hart lab, PICKLES (Pooled In-Vitro CRISPR Knockout Library Essentiality Screens), integrates multiple CRISPR library results to determine the essentiality of each cell line for a specific gene product. The results of multiple CRISPR libraries are merged, and the essentiality of each cell line for a particular gene product is ranked by a score called BF (Bayes Factor), which can be searched [62]. For example, *ERBB2*, which is often amplified in breast cancer, is essential for survival and proliferation in the breast cancer cell line MDAMB453, esophageal cancer cell line KYSE410, etc., while it is not essential in the pancreatic cancer cell line MIAPAca2, according to the ANOVA Dataset of PICKLES. However, in the shRNA library dataset (SCORE), it is also possible to compare and integrate with the CRISPR library dataset. High essentiality is an essential factor for cell line survival, and KO tends to make the cell line unable to survive and grow. In addition, cell lines can be sorted and ranked in the tabs of cancer type, mutation, copy number, and expression, and they can be browsed by a dot. The ability to examine whether the candidate genes identified in the CRISPR KO library can affect the survival and proliferation of the target cell line in PICKLES can greatly reduce the time and effort required for candidate gene selection experiments. In the recently updated version, it is also possible to refer to the co-essentiality of primary and secondary genes, which is a great help in narrowing down multiple candidate genes.

Recently, iCSDB, an integrated database of CRISPR screens, has integrated DepMap with BioGRIS ORCS, a database of CRISPR screening results from PubMed articles, to enable more clinical searches. We integrated DepMap and BioGRIS ORCS—databases of CRISPR screening results from PubMed articles—to enable a more clinical search. In addition, clinical and molecular data were added as annotated data for easier searching than cell lines [63].

## 5. Use of Database Focusing on Synthetic Lethality

**Figure 2:**
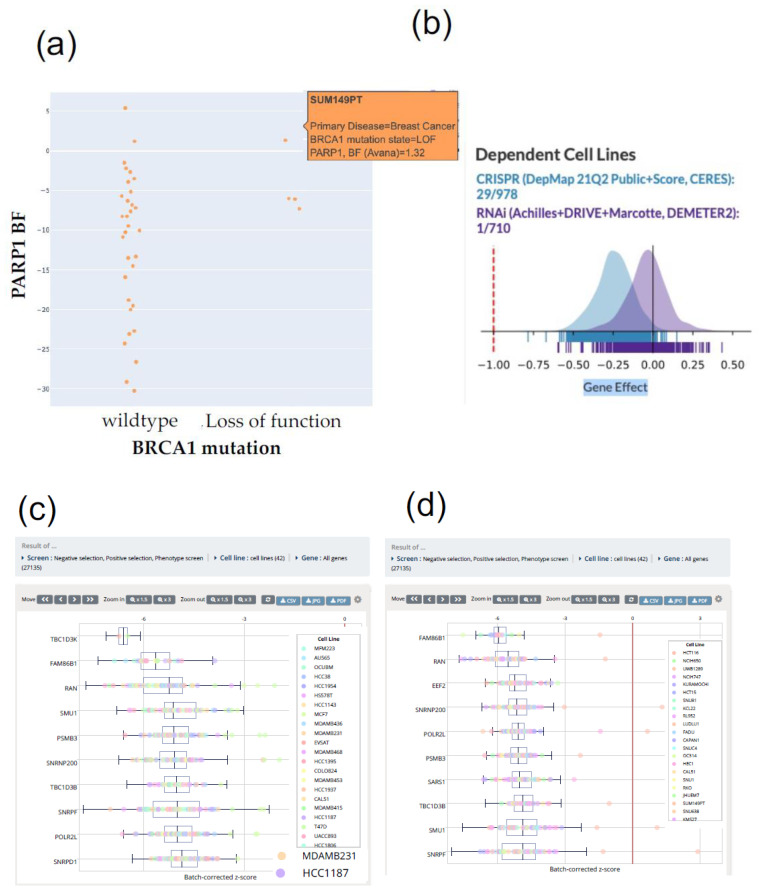
CRISPR library database. (**a**) Result of BRCA1 mutation and PARP1 BF from PICKLES (http://pickles.hart-lab.org (accessed on 24 October 2021)). *BRCA* loss-of-function cell lines are dependent on *PARP1* compared with *BRCA* wild-type cell lines. BF: Bayes factor (**b**) PARP1-dependent cell line from Depmap portal (https://depmap.org/portal/ (accessed on 24 October 2021)). Depmap’s “Dependent Cell Lines” category suggests that a chosen gene is a ‘common essential’ or ‘strongly selective’ judgment for cell lines regarding CRISPR and RNAi database. Because searching ‘*PARP1*′ in Depmap showed no judgment in the “Dependent Cell Lines” category, PARP1 is not an essential gene for most cell lines and not-selective drug targets. (**c**) Top essential genes of the breast cancer cell lines from iCSDB (https://www.kobic.re.kr/icsdb/ (accessed on 24 October 2021)). The result of the top 10 essential genes of the 42 breast cancer cell lines in iCSDB. The genes essentiality score is depicted with a boxplot. Colored dot points each cell line, annotated in square right side (for example: orange dot MDAMB231 cell line, purple dot HCC1187 cell line). (**d**) Top essential genes of the *BRCA1/2* deleted cell lines from iCSDB (https://www.kobic.re.kr/icsdb/ (accessed on 24 October 2021)). The result of top 10 essential genes of the 42 *BRCA1/2* deleted cell lines (not organ-specific) in iCSDB. The genes essentiality score is depicted with a boxplot. Colored dot points each cell line, annotated in square right side.

To assess the co-essentiality of interesting genes in cell lines, PICKLES is effective. For example, on the top screen of PICKLES, check “Breast cancer” in the “Cancer type” field, enter “PARP1” in the “Primary gene” field, and “BRCA1” in the “Secondary gene” field (the results of mutation and copy number are reflected in the Secondary gene). In the Co-essentiality tab, PARP1 BF and BRCA1 BF are negatively correlated (Figure 2a). In the Mutation tab, PARP1 BF and BRCA1 BF are negatively correlated in the *BRCA1* deletion and mutation groups. In the mutation group, the PARP1 BF exceeded that of the BRCA1 wild type. These results indicate that PARP1 is highly dependent on *BRCA1* deletion mutations in breast cancer cell lines and that PARP1 may be a good target for synthetic lethality in *BRCA1* mutant lines.

In the DepMap portal, when PARP1 is entered, CRISPR (DepMap 21Q2 Public + Score, CERES) is 29/978 in the Dependent cell line, indicating that it is not Common essential (Figure 2b). In Target Tractability, Bioactive Compounds, Druggable Structure, Druggable by Ligand Based Assessment, and Enzyme were all judged to be Yes, indicating that they are useful targets for inhibitors.

The iCSDB is useful for searching by cell line, and the Choose by cell line annotation in the Cell line selector section allows you to select a cell line. For example, if you select HCC1187 and MDAMB231 cells, which are triple-negative cell lines in the breast cancer cell line, and click the “Search CRISPR screen,” the essential genes in both cell lines will be displayed in boxplot order (Figure 2c). However, since this result includes the essential genes required in many cell lines, it is necessary to narrow down the essential genes by referring to databases such as PICKLES. Furthermore, in iCSDB, it is possible to search for gene mutations in all cell lines registered. For example, in Cell line selector, check “choose by molecular characteristics from CCLE” and “Mutation” tabs, input “BRCA” in “Gene Name”. Then, sort by “Mutation Type”, and choose cell lines as described “Frame_Shift_Del” and “Nonsense_Mutation” (Figure 2d). We can see the essential genes in all cell lines with a BRCA deletion.

In this way, virtual CRISPR screening can be used to narrow down and validate candidate genes using the database while conducting our screening. However, the data in the database are obtained from cancer-derived cell lines, and information on essentiality in normal tissues will be required for clinical application, so the question is how to apply CRISPR library screening.

A method for performing CRISPR screening across numerous cell lines by assigning gene barcodes has also been established, allowing genome-wide analysis of numerous cells at once and linking to drug information. PRISM is a system that can screen more than 10,000 compounds in more than 1000 different cell lines at once by introducing genetic barcodes into the cell lines [66]. Yifeng Xia et al. developed BMS-PRISM using a plasmid incorporating the PRISM barcode and Cas9 and developed a system for CRISPR screening across cell lines at once [67]. They used EGFR sgRNA and its inhibitor Elrotinib to screen 368 cell lines transfected with gene expression barcodes and Cas9 for EGFR Essentiality at one time. In all cases, we can extract cell lines with high EGFR Essentiality, which correlated well with Depmap, a database described above. Thus, combining the PRISM and CRISPR library, which can screen numerous cell lines at once, can efficiently confirm the essentiality. Furthermore, information on gene mutations in cancers is available in TCGA, and more efficient identification of molecular targets can be accelerated with combination analysis.

## 6. Conclusions

In this study, we reviewed the principles of the CRISPR-Cas9 system, types of CRISPR libraries, methods, databases, and research results. The database has been renewed and reborn daily. In the future, the database only, without using CRISPR screening for cell lines, will make us discover new candidate genes for synthetic lethal targets of cancer. As databases become enriched, CRISPR screening conditions will be required for the more specific ingenuity mentioned above. In addition, there is limited data on CRISPR inhibition and activation compared to knock-out screenings.

In the future, culturable tissues, such as organoids, will be generated from cancer tissues collected from patients, and small-sized but efficient CRISPR library screening will be used to discover individual therapeutic targets, including synthetic lethal targets for cancer, which is expected to be the ultimate precision medicine. By consolidating these data and updating the database, an era in which humanity can truly conquer cancer will arrive.

## Figures and Tables

**Table 1:** CRISPR knockout library.

Name	Advantage	sgRNAS/Gene	Total gRNAs	Ref.
Garnett Lab MinLibCas9 Library	Minimal genome-wide human CRISPR-Cas9 library	2	37,722	[20]
Human CRISPR Knockout Pooled Library (Gattinara)	Minimal genome-wide human CRISPR-Cas9 library compatible with the Brunello library	2	40,964	[21]
Human GeCKO v2	Targets early consecutive exons Contains 1000 control (non-targeting) sgRNAs	3 or 6	123,411	[22]
Broad GPP genome-wide Brunello	Improved on-target activity predictions and off-target scores compared to the GeCKOv2 library	4	76,441	[23]
Human genome-wide library v1	Targets sites in a region close to the translation initiation site for complete gene disruption	4	77,406	[24]
Human improved genome-wide library v1	gRNAs redesigned using pipeline with a new design Improved on-target sensitivity and reduced off-target effect scaffold	5	90,709	[25]
Human genome-wide reduced double-gRNA library	Optimization of guide RNA designs and delivery of two gRNAs with each construct	3	59,576	[26]
Human whole genome sgRNA iBAR library	Incorporates four 6-basepair internal barcodes (iBARs) in each sgRNA Efficient and accurate screening at high MOI	3	58,630	[27]
Mini-human AsCpf1-based human genome-wide knockout library	Each gene targeted by an AsCpf1(AsCas12a)-based array containing 3–4 guides concatenated in one vector	3–4	17,032	[28]
Toronto KnockOut (TKO) version3	Improved accuracy, efficiency, and scalability for CRISPR screens compared to TKO version 1	4	70,948	[29]

**Table 2:** CRISPR activation and inhibition library.

Name	Advantages	sgRNAs/Gene	Total gRNAs	Ref.
**Activation**				
CRISPRa-v2	SunTag-VP64 activation system	5 or 10	104,540 or 209,080	[30]
SAM (Synergistic Activation Mediator) v1–3 plasmid system	Comprises three plasmids (Cas9-VP64 fusion, gRNA incorporating two MS2 RNA aptamers at the tetraloop and stem-loop 2, and MS2-P65-HST) Efficient gene upregulation	3	70,290	[13]
SAM v2–2 plasmid system	Comprises two plasmids (gRNA library–lenti SAM v2 backbone and MS2-P65-HST) Efficient gene upregulation	3	70,290	[31]
Human CRISPR lncRNA activation pooled library	SAM library for transcriptional activation of lncRNAs	10	96,458	[32]
Broad GPP activation Calabrese p65-HSF	Modified tracrRNA with two MS2 loops and two PP7 loops Better concordance of sgRNAs compared to the SAM v2 library	3 or 6	56,762 (Set A) 56,476 (Set B)	[23]
**Inhibition**				
CRISPRi-v2	dCas9-KRAB represses TSS downstream of TSS sites	5 or 10	104,535 209,070	[30]
Broad GPP inhibition Dolcetto	gRNAs redesigned based on FANTOM5 CAGE data Gene regulation equal to the CRISPR KO library	3 or 6	57,050 (Set A) 57,011 (Set B)	[23]

**Table 3:** CRISPR screening for synthetic lethal research.

Cancer Type (Cell Line)	Altered Gene/Drug	CRISPR Type	Library	Synthetic Lethal Hits	Ref.
Colorectal cancer (HCT116)	KRAS (G13D)	Knockout	GeCKOv2	NADK, KHK, INO80C	[39]
Pancreatic cancer (HPAF-II)	RNF43	Knockout	TKO	FZD5, Wnt pathway genes	[40]
Lung squamous cell carcinoma (H226 shp63)	ΔNp63α	Knockout	GeCKOv2	RHOA, TGFBR2	[41]
Small-cell lung cancer (NCI-H82)	RB1−/−	Knockout	Custom	Aurora kinase B	[42]
Hepatocellular carcinoma (PLC/PRF/5)	ATRX loss	Knockout	GeCKOv2	WEE1	[43]
Chronic myelogenous leukemia (K562)	–	Double knockout	Paired sgRNA	BCL2L1–MCL1 combination	[44]
T-acute lymphocytic leukemia (CCRF-CEM)	Asparaginase-resistant	Knockout	GeCKO	NKD2, LGR6, ASNS	[45]
Pancreatic cancer, non-small-cell lung cancer (CFPAC-1, A549, NCIH23)	MEK1/2 inhibition	Knockout	Avana-4 barcoded sgRNA	SHOC2	[46]
Colorectal cancer, breast cancer (HCT116, MCF10A)	ATR inhibition	Knockout	TKOv3	RNASEH2	[47]
Triple-negative breast cancer (SUM159, SUM149)	BET bromodomain inhibitor	Knockout	H1 and H2	CDK4 and BRD2	[48]
Murine acute myelogenous leukemia (RN2)	–	Cas12a (Cpf1) multigene knockout	Custom	BRD9 & JMJD6, KAT6A & JMJD6, BRPF1 & JMJD6	[49]
Osteosarcoma (U2)	GPX4 (ferroptosis-resistant)	Knockout	Custom	FSP1 (AIFM2)	[50]
Myc-driven breast cancer model (MYC-ER HMECs)	MYC	Knockout	RNA-binding protein pooled CRISPR knockout	YTHDF2	[51]
Colorectal cancer (BRCA2−/− DLD1)	BRCA2 mutation	Knockout	Custom	FEN1, APEX2	[52]
Pancreatic cancer (PANC-1)	Gemcitabine	Knockout	Brunello	PSMA6	[53]
Pancreatic cancer (PATU8902)	Trametinib	Knockout	GeCKOv2 Avana	CIC, ATXN1L	[54]
Pancreatic cancer (PDX366)	MEK and CENPE inhibitor	Knockout	Nuclear proteins gRNA sub-pool	CENPE, RRM1	[55]
Pancreatic cancer (Mia PaCa-2, A2780)	Gemcitabine, NUC-1031	Knockout	GeCKOv2	DCK, DCTPP1	[56]
Glioblastoma stem-like cells (2 patient-derived cells)	EGFR+PI3K signaling	Knockout	GeCKOv1	PKMYT1, WEE1	[57]
Primary human retinal pigment epithelial cells (RPE1-hTERT p53−/− Flag-Cas9 cells)	27 DNA-damaging agents	Knockout	TKO v2 TKO v3	ERCC6L2, TOP2, ELOF1, STK19	[58]

**Table 4:** CRISPR screening database.

Database	Characteristics	Number of Cell Lines	Usage	Ref.	URL
DepMap portal	Integrates CRISPR KO screening databases (DepMap, Sanger, and GeCKO) and unifies cellular model (CCLE) and drug sensitivity (PRISM) databases	786 cell lines 42 cancer types	Discovering genetic and pharmacological dependenciesPrioritizing tumor contexts and predictive biomarkers Exploring over 2000 cancer models	[60]	https://depmap.org/portal/ (accessed on 24 Octorber 2021)
Project Score	Genetic screens for identifying cancer dependencies	914 cell lines 25 tissues 7470 fitness genes	Investigating specific genes, cancer cell models, or tissue types Browsing all gene fitness scores	[61]	https://score.depmap.sanger.ac.uk/ (accessed on 24 Octorber 2021)
PICKLES (Pooled In-Vitro CRISPR Knockout Library Essentiality Screens)	Cell line essentiality profiles for CRISPR KO library and shRNA datasets	More than 50 cell lines for CRISPR screening and 100 cell lines for shRNA library	Easily exploring cell line essentiality and co-essentiality	[62]	http://pickles.hart-lab.org (accessed on 24 Octorber 2021)
iCSDB	Integrated DepMap portal and BioGRID ORCS Integrated database of CRISPR-CAS9 screening experiments for human cell lines and clinical information	976 cell lines	Easily searching for cell line data associated with clinical and molecular data	[63]	https://www.kobic.re.kr/icsdb/ (accessed on 24 Octorber 2021)
CRISP-view	Data from 167 studies collected from PubMed, Gene Expression Omnibus (GEO), and Ensemble and Cancer Cell Line Encyclopedia (CCLE)	321 human samples 825 mouse samples	Web interface visualizing datasets, allowing the exploration of interesting genes, cell lines, tissues, studies, or conditions	[64]	http://crispview.weililab.org (accessed on 24 Octorber 2021)

## Data Availability

No data were used in this review.
